# The Relation Between Extracellular Vesicles Released From Red Blood Cells, Their Cargo, and the Clearance by Macrophages

**DOI:** 10.3389/fphys.2022.783260

**Published:** 2022-03-31

**Authors:** Duc Bach Nguyen, Hanh Triet Tran, Lars Kaestner, Ingolf Bernhardt

**Affiliations:** ^1^Department of Molecular Biology, Faculty of Biotechnology, Vietnam National University of Agriculture, Hanoi, Vietnam; ^2^Division of Aquacultural Biotechnology, Biotechnology Center of Ho Chi Minh City, Ho Chi Minh City, Vietnam; ^3^Theoretical Medicine and Biosciences, Medical Faculty, Saarland University, Homburg, Germany; ^4^Dynamics of Fluids, Experimental Physics, Saarland University, Saarbruecken, Germany; ^5^Laboratory of Biophysics, Faculty of Natural and Technical Sciences, Saarland University, Saarbruecken, Germany

**Keywords:** human red blood cells, macrophages, THP-1 cells, cell-based vehicles, extracellular vesicles, clearance

## Abstract

Extracellular vesicles (EVs) are cell-derived membrane particles that include exosomes, ectosomes, microvesicles, microparticles, apoptotic bodies, and other EV subsets. EVs are involved in intercellular communication and the transport of macromolecules between cells. Here, we propose and test the ability of red blood cell (RBC)-derived EVs (RBC-EVs) as putative drug carriers. EVs were produced by treating RBCs with Phorbol-12-myristate-13-acetate (PMA) and separating from the cells by differential centrifugation steps. RBC-EVs were characterized by size determination, flow cytometry, and scanning electron microscopy (SEM). EVs were loaded with DNA plasmids coding for the green fluorescent protein (GFP) by electroporation. The DNA-loaded EVs (DNA-EVs) were used to transfect THP-1-derived macrophages and analyzed by fluorescence microscopy and flow cytometry. The results showed that RBC-EVs had an almost spherical shape and a polydispersity in their size with an average of 197 ± 44 nm and with a zeta potential of −36 ± 8 mV. RBC-EVs were successfully loaded with DNA but associated with an increase of the polydispersity index (PdI) and showed a positive signal with Picogreen. DNA-EVs were almost completely taken up by macrophages within 24 h, however, resulting in the expression of the GFP in a subpopulation of macrophages. As the way, we designed that RBC-EVs could be potential nucleic acid carriers when the immune system was addressed. This study may contribute to the understanding of the role of EVs in the development of microvesicle-based vehicles.

## Introduction

In 1987, extracellular vesicles (EVs) were firstly described in erythropoietic cells by Johnstone et al. (1987) when cultured sheep reticulocytes *in vitro*. Later EVs were found released from blood cells, body fluids of animals, and also from bacteria and plant cells (Akuma et al., 2019; Macia et al., 2020). Under physiological and pathological conditions, various cell types secrete EVs (Raposo and Stoorvogel, 2013; Lötvall et al., 2014; Antonyak and Cerione, 2015; Witwer et al., 2017; Van Niel et al., 2018; Murphy et al., 2019; Thangaraju et al., 2020). Recently, all secreted membrane-enclosed vesicles are collectively called EVs, which include exosomes, ectosomes, microvesicles, microparticles, apoptotic bodies, and other EV subsets (Lötvall et al., 2014; Witwer et al., 2017).

Red blood cell (RBC)-derived EVs (RBC-EVs) are secreted during erythropoiesis, cellular aging, such as stored blood (Bosman et al., 2008; Kriebardis et al., 2008; Thangaraju et al., 2020), or in response to activated conditions, such as an increase of intracellular Ca^2+^ or activation of protein kinase C (PKC) (Nguyen et al., 2016, 2017; Bernhardt et al., 2020), and disease conditions (Alaarg et al., 2013; Mantel et al., 2013; Leal et al., 2018). Under physiological and pathological conditions, RBC-EVs could be loaded with proteins, lipids, and miRNAs might be vital for homeostasis. RBC-EVs are also involved in the communication between RBCs and endothelium to regulate NO and O_2_, redox balance, immunomodulation, and the pro-coagulant effects in several disease states (Van der Meijden et al., 2012; Thangaraju et al., 2020). In addition, RBC-EVs are involved in inducing the secretion of proinflammatory cytokines and chemokines (Danesh et al., 2014; Zecher et al., 2014).

Recently, RBC-EVs have received much attention in respect to intercellular communication (Nguyen et al., 2016; Noubouossie et al., 2020). It has been reported that RBC-EVs can be applied for RNA delivery in gene therapy (Usman et al., 2018). As the number of RBCs exceeds that of all other cell types, therefore, the number of EVs released from RBC is myriad in the whole blood (Wu et al., 2017). Current theories mainly focus on the removal of senescent RBCs and their EVs as a part of the homeostasis governed by macrophages (de Back et al., 2014). Therefore, the understanding and application of RBC-EVs as novel vehicles for drugs delivery or treatment is of utmost importance. So far, limited data are available about using RBC-EVs as vehicles for the transport of macromolecules and also the knowledge about the fate of RBC-EVs in circulation is not available. In addition, the information about the loading of macromolecules into RBC-EVs and the delivery of loaded RBC-EVs are limited.

In the last decades, the development of nanoparticle-based vehicles has improved the therapeutic efficacy of certain drugs by allowing controlled access and administration to target cells. However, several problems associated with synthetic nanoparticles have been observed, such as cell cytotoxicity, immunogenicity, rapid elimination by the phagocytic mononuclear system, and the specific sites of action (Lee et al., 2012; Murphy et al., 2019; Gutierrez-Millan et al., 2021). Because EVs possess nanoscale dimensions and a variety of adhesion proteins presenting on their surface that support the specific interaction with targeted cell types, they are qualified as potential vehicles for drug delivery (Maas et al., 2017; Bunggulawa et al., 2018; Elsharkasy et al., 2020; Gutierrez-Millan et al., 2021). Therefore, EV can be a means of transport for drugs or macromolecules open a new approach for targeted drug delivery and treatment (Kanada et al., 2015; Balachandran et al., 2019; Li C. et al., 2019; Li X. et al., 2019; Butreddy et al., 2021; Gutierrez-Millan et al., 2021). Recent studies have proposed that EVs play a role in intercellular communication and transport molecules, such as mRNA, miRNA, and proteins between cells (Chitoiu et al., 2020; Gurung et al., 2021).

Within this study, we produced RBC-EVs, characterized them physically, and loaded them with DNA. Furthermore, we investigated the ability of EVs to carry DNA and the interaction between EVs and macrophages differentiated from THP-1 cells. The study contributes to the understanding of RBC-EVs properties and encourages the further development of microvesicle-based vehicles as a new approach for targeted drug delivery and treatment (such as gene therapy). The study also suggests that the macrophages may limit the applicability of RBC-EVs *in vivo*.

## Materials and Methods

### Blood and Solutions

Fresh human venous blood from healthy donors was withdrawn by venipuncture into citrate-coated tubes or with heparin as an anticoagulant, kept at 4°C, and used on the same day. Fresh whole blood was centrifuged at 2,000 g for 5 min at room temperature and the plasma, and buffy coat was removed by aspiration. Subsequently, RBCs were washed 3 times in N-2-hydroxyethylpiperazine-N′-2-ethanesulfonic acid (HEPES)-buffered solution (HPS) containing (mM): NaCl 145, KCl 7.5, glucose 10, HEPES 10, pH 7.4, and finally, RBCs were resuspended in HPS and kept at 4°C for experiments.

### Stimulation of Red Blood Cells and Isolation of Extracellular Vesicles

Red blood cells were suspended in HPS at a hematocrit of 0.5% in the presence of 2 mM CaCl_2_. Phorbol 12-myristate 13-acetate (PMA, Sigma-Aldrich, St. Louis, United States) was added at a concentration of 6 μM and the cell suspension was incubated for 2 h at 37°C with occasional shaking. Subsequently, cell suspensions were subjected to differential centrifugation. First, a centrifugation step at 1,500 g for 10 min was applied followed by centrifugation at 3,000 g for 15 min twice to remove intact cells, cell debris, and apoptotic blebs. The supernatants were collected and further centrifuged at 25,000 g for 1 h at 4°C to harvest large size EVs. Subsequently, the collected supernatants were transferred to new tubes and ultra-centrifuged at 35,000 rpm (corresponding to ∼ 200,000 g) for 2 h at 4°C for the isolation of small size EVs (Beckman Coulter, Swinging Bucket rotor SW40 Ti, k-factor 137). Pellets obtained after each centrifugation step were resuspended in Milli-Q water (ultra-pure) for morphological, size, and zeta potential measurements (Nguyen et al., 2016).

### Loading of DNA Plasmids Into Extracellular Vesicles

DNA plasmid (pEGFP-C3, Clontech, United States) was transformed into competent *Escherichia coli* DH5α cells and cloned in the DH-5 alpha competent cells. It was cultured overnight on lysogeny broth (LB) agar medium (22700025, Thermo Fisher Scientific, United Kingdom) containing a final concentration of 30 μg/ml kanamycin. The resulting colonies were individually selected and cultured in LB liquid medium (12780052, LB Broth Base, Thermo Fisher Scientific, United Kingdom) shaken at 37°C for 16 h. The plasmid was extracted using a Plasmid Midi Kit (12143, Qiagen, United Kingdom). Purified DNA plasmid DNA quantification using a NanoDrop^®^ (ND-1000 UV-Vis spectrophotometer, Thermo Fisher Scientific, United Kingdom).

In this study, DNA plasmid (dsDNA) was loaded into small size EVs by electroporation. To load DNA into EVs, a 10 μl DNA plasmid (1.0 μg/μl) aliquot was added into 1 ml freshly isolated EVs [suspended in phosphate-buffered saline (PBS) buffer, containing (mM): (NaCl 137.0, KCl 2.7, Na_2_HPO_4_ 10, KH_2_PO_4_ 1.8, CaCl_2_.2H_2_O 1.0), pH 7.4] at a concentration of about 10^7^ particles/ml. DNA plasmid and EVs were mixed throughout gently by pipetting. The mixture of EVs and DNA was prepared as described and transferred (200 μl) to the electroporation cuvettes (T-25714-02, Eppendorf, United Kingdom) gap width 2 mm, then electroporated for 5.0 ms at 2,000 V (Multiporator 36205-10, Eppendorf^®^ Electroporation Systems, United Kingdom). The electroporation was carried out for several batches to obtain enough DNA-loaded EVs (DNA-EVs) for experiments. The efficiency of the transformation of DNA plasmid into EVs was investigated by flow cytometry with specific fluorescent dyes for (dsDNA).

### THP-1 Cell Culture

The human monocytic leukemia cell line, THP-1, was grown in Roswell Park Memorial Institute (RPMI) 1640 medium supplemented with 10% Fetal Bovine Serum (FBS, Gibco™, Thermo Fisher Scientific, United Kingdom), 10 mM HEPES (#15630-056, Gibco™, Thermo Fisher Scientific, United Kingdom), 1% L-glutamine, and 50 μg/ml of cefotaxime (Sigma, St. Louis, United States)/ml. THP-1 monocytes are differentiated into macrophages following a modified procedure by incubation with 100 nM PMA for 24 h (Genin et al., 2015). After washing in PBS to remove PMA, cells were transferred to PMA-free RPMI 1640 medium for 48 h resting. Macrophage M2 polarization was obtained by incubation with interleukin 4 (20 ng/ml) for 48 h. After being washed in PBS twice, differentiated THP-1 cells were seeded at a density of 5.105 cells/well in 24-well plates and cultured for 24 h in RPMI media containing 10% FBS, and 50 μg/ml of cefotaxime before transfection with EVs.

### Transfection Experiments

The differentiated THP-1 cells were infected with DNA-EVs treated or untreated with annexin V. For each well, the same amount of DNA-EVs (∼10^7^ EVs/μl) was added to the cell culture. The remaining EVs in cell culture were observed by fluorescence microscopy, DNA-EVs were loaded with propidium iodide (PI) (stock solution of 1 mg/ml in distilled water). The remaining EVs after 0, 6, 12, 18, and 24 h of transfection were also analyzed by flow cytometry. The suspensions containing EVs were collected by centrifugation at 10,000 g for 10 min at 4°C to remove cells. The concentration of EVs in solutions was measured by flow cytometry with 100,000 events using the following parameters: (i) the number of events in the gate of EVs, and (ii) the volume of sample used in the counting process. The number of EVs remaining in the solution reflected the uptake of differentiated THP-1 cells during the transfection. Data are presented as percent of remaining EVs relative to the controls.

### Cell Viability (Trypan Blue Assay)

THP-1 monocytes were seeded at 180,000 cells/well in 24 well plates and differentiated in macrophages as described. Before being transfected with EVs, cells were tested for viability using trypan blue. In short, cells were detached from the flask by trypsinization and centrifuged the cell suspension for 5 min at 1,000 g (Eppendorf 5415R, Germany). Subsequently, cells were resuspended in 1 ml of culture medium using a pipette to obtain a single-cell suspension. In total, 100 μl of trypan blue solution (0.4%) was added to 100 μl of cell suspension and incubated for 5 min to stain the dead cells. The cells were counted using a hemocytometer and a light microscope (Eclipse Ei, Nikon, Japan). The percentage of viability and number of cells in the culture were calculated by considering the final dilution factor (Piccinini et al., 2017).

### Flow Cytometry

Samples were analyzed using a Beckman-Coulter FC500 cytometer (High Wycombe, United Kingdom). For each sample, a minimum of 20,000 events were analyzed. All acquisition and analysis were carried out in log mode. The parameters were set up using a standard calibration kit (BD Calibrite™, BD Calibrate 3 Beads, BD Biosciences, United States). Parameters of both forward and side scatter were adjusted to remove instrument noise (dust). The gating process was carried out using a combination of sheath fluid (blank), samples of stimulated RBCs, isolated EVs stained with the fluorescent probes annexin V-FITC for phosphatidylserine (PS), and Dil stain {1,1′-Dioctadecyl-3,3,3′,3′-tetramethylindocarbocyanine Perchlorate [′DiI′; DiIC18(3)]} as the lipophilic tracer. Freshly washed RBCs were used in the calibration and gating to localize the noise events. The concentration of EVs (as number of EVs/ml) was calculated based on the gating and counting the number of events showing a positive signal with annexin V-FITC and Dil stain in the gate of EVs.

### Morphological Analysis Using Scanning Electron Microscopy

RBCs stimulated by PMA were fixed with 2% glutaraldehyde at room temperature for 10 min and washed by PBS-TWEEN^®^ solution (1 tablet per litter to yield 140 mM NaCl, 3 mM KCl, 0.05% TWEEN 20 detergent, 10 mM phosphate buffer, pH 7.4 at 25°C) (PBS-TWEEN^®^ tablets, Calbiochem- Merck, Darmstadt, Germany) to remove glutaraldehyde by centrifugation at 5,000 g for 5 min at room temperature. The pellets were resuspended in Milli-Q water and immediately applied on glass slides and air-dried for 1 h. The slides were dipped quickly and gently washed stepwise with ethanol from 50, 70, and 90–100% for dehydration. For SEM analysis, the prepared slides were sputtered with a gold layer of 15 nm thickness before SEM imaging (Sputter coater: Quorum Q150R ES, Quorum Technologies Ltd., East Grinstead, United Kingdom) and kept in a closed box at room temperature.

To prepare samples of EVs for SEM analysis, 0.5 ml EVs (about 10^7^ particles/ml) were fixed with glutaraldehyde at a final concentration of 2% for 5 min at room temperature. Subsequently, the samples were washed in PBS-TWEEN solution by an ultracentrifuge step at 200,000 g for 30 min at 4°C. Further steps were performed similarly to the preparation of stimulated RBCs described above. For SEM imaging (EVO HD15, Carl Zeiss Microscopy GmbH, Jena, Germany), several randomly selected frames from each sample were captured for morphological observation and statistical purposes. SEM imaging was carried out using a 5 kV acceleration voltage and a secondary electron (SE) detector.

### Size and Zeta Potential Measurement

Zetasizer Nano ZS (Malvern, Worcestershire, United Kingdom) was used for EVs size and zeta potential measurement. Uniform polystyrene particles of 100, 200, and 400 nm diameter (Bangs Laboratories, Thermo Fishers Scientific, United States) at 0.01% in PBS were used to verify instrument operation. For size measurement, the EV samples were diluted in Milli-Q water (attenuator index position in the range from 7 to 9) and analyzed using the standard operation procedure (SOP) as follows: sample refractive index 1.43 (phospholipid liposomes), dispersant refractive index 1.33 (water), system temperature at 25°C, and sample equilibration time for 60 s. One millimeter of each sample was measured in disposable polystyrene (DTS0012, Malvern Instruments, Worcestershire, United Kingdom) with a path length of 10 mm. Observed populations of particles were characterized by associated Z-average size (nm) and polydispersity index (PdI).

For zeta potential measurement, the disposable capillary cell DTS1070 (Malvern Instruments, United Kingdom) was used. Samples were measured in PBS-TWEEN solution. The SOP was set up similarly as described above. Each sample was measured 3 times with a maximum of 100 runs in automatic mode. Observed populations of particles were characterized by associated phase (rad) and zeta potential (mV). The Malvern Zetasizer software v7.03 was used to collect and analyze the data.

### Statistics

All experiments were repeated at least three times. Data were presented as the mean values ± SD of at least 3 independent experiments. Statistical analysis was performed using Student’s *t*-test when Gaussian distributed. Differences were considered significant when *p* < 0.05 or *p* < 0.01, otherwise, a Mann-Whitney test was performed.

## Results

### Stimulation, Isolation, and Characterization of Red Blood Cell-Extracellular Vesicles

**FIGURE 1:**
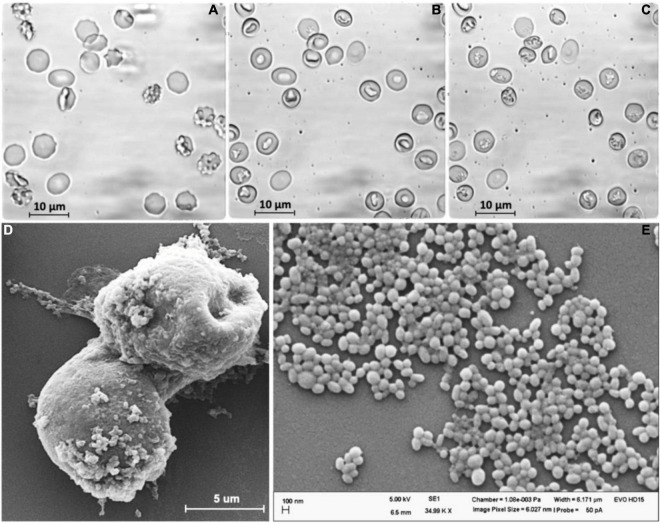
Extracellular vesicles (EVs) were released from human red blood cells (RBCs) stimulated by PMA (6 μM) after **(A)** 0 min, **(B)** 15 min, and **(C)** 60 min. **(D)** SEM analysis of stimulated RBCs treated by PMA after 60 min. **(E)** EVs released from RBCs.

To produce adequate RBC-EVs, RBCs (0.5% hematocrit) were treated with PMA as described. After 10 min of PMA treatment in the presence or absence of Ca^2+^, RBCs were deformed (Figure 1A) and changed their shapes to stomatocytes (Figure 1B). The stomatocyte shape was stable and correlated with the formation and shedding of EVs on the surface of the RBCs. The formation of EVs could be observed under a transmission light microscope after 10 min of stimulation, and the number of EVs was increased over time (Figure 1C). The stimulated RBCs and EVs adhered together to form a clot with EVs extruding on the outer surface (Figure 1D). SEM analysis showed that the EVs released from RBCs had an almost spherical shape and polydispersity in their size. Statistical analysis revealed that the size of EVs was in the range of 100–300 nm at an average value of about 200 nm (Figure 1E).

**FIGURE 2:**
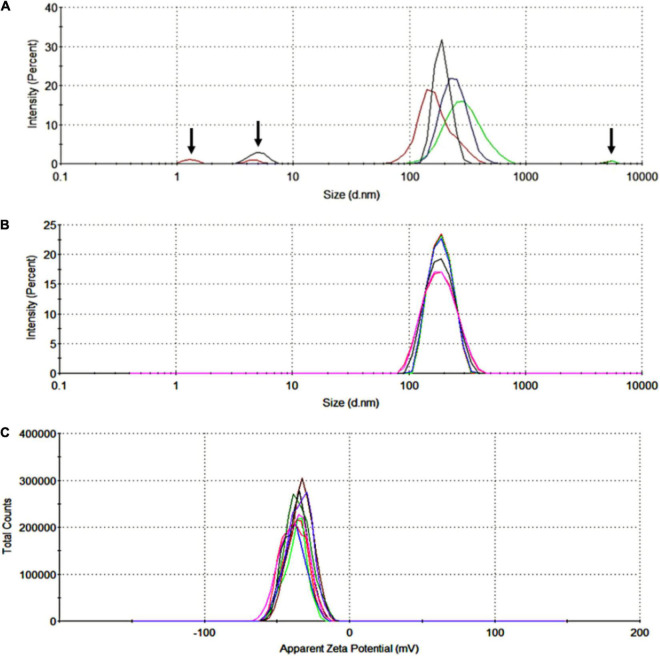
Size distribution and zeta potential of red blood cell-derived extracellular vesicles (RBC-EVs) stimulated by PMA. **(A)** The highly heterogeneous in size of RBC-EVs, the arrows indicated large particles (cell debris or apoptotic blebs) and fine particles (dust or noise), **(B)** size distribution by the intensity of EVs collected in the fractions of ultra-centrifuged at 200,000 g for 2 h (overlay of 6 separated measurements), **(C)** zeta potential distribution of EVs (overlay of 10 separated measurements).

The EVs were collected by differential centrifugation as described above. After 60 min stimulated by PMA, the cell suspension was centrifuged at 1,500 g for 10 min to collect the EVs and remove intact cells. Size distribution analysis showed high heterogeneity in size in this fraction (Figure 2A). Size distribution of the fractions of EVs was ultracentrifuged at 200,000 g for 2 h at 4°C that showed an average value of 196.6 ± 43.4 nm (Figure 2B). Zeta potential analysis of EVs suspended in Milli-Q water (ultra-pure) showed a negative charge with an average value of −36.4 ± 7.8 mV (Figure 2C).

### Loading Red Blood Cell-Extracellular Vesicles With DNA

**FIGURE 3:**
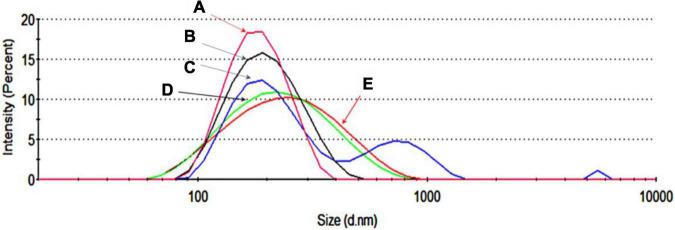
Size distribution by the intensity of extracellular vesicles (EVs) under different conditions. **(A)** EVs in PBS-TWEEN solution. **(B)** Electroporated EVs without DNA. **(C)** Electroporated EVs with DNA. **(D)** DNA dissolved in MQ water. **(E)** Electroporated DNA without EVs.

The next step was to load the RBC-EVs with DNA plasmids by electroporation as described in “Materials and Methods” section. These DNA-EVs were investigated by the NanoSizer and by flow cytometry. For the NanoSizer analysis, the size distributions of RBC-EVs under different conditions were investigated. The effect of electroporation changed the size of EVs in the presence and absence of DNA plasmid (cp. Figures 3A,B) and also the DNA plasmid itself (cp. Figures 3D,E). There was a significant change in the size distribution of EVs after electroporation in the presence of DNA plasmid. The population of EVs or DNA-EVs included sub-populations with sizes in the ranges of 200, 700, and 6,000 nm (Figure 3C).

**FIGURE 4:**
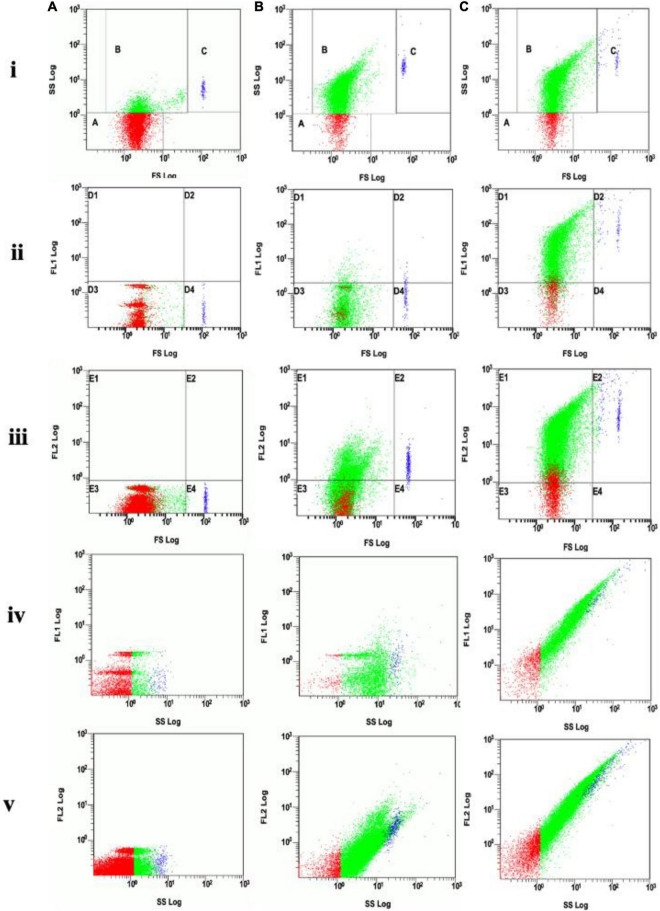
**(A)** Analysis of EVs without electroporation (control). **(B)** Analysis of DNA-electroporated extracellular vesicles (EVs) stained with lipophilic Dil fluorescent dye, the EVs showing positive fluorescence signal was distributed in gates E1 and E2 of the FL2 channel. **(C)** Analysis of DNA-electroporated EVs double-stained with both Picogreen and lipophilic Dil fluorescent dyes. EVs showing positive fluorescence signal distributed in gates D1 and D2 in the FL1 channel (for Picogreen) and in gates E1 and E2 in the FL2 channel (for Dil). On each row, **(i)** the forward scatter (FS) vs. side scatter (SS), gate A containing dust/noise, gate B containing EVs and gate C containing large or aggregated EVs, **(ii)** FL1 vs. FS, **(iii)** FL2 vs. FS, **(iv)** FL1 vs. SS, and **(v)** FL2 vs. SS.

Flow cytometry analysis showed that electroporation was an effective approach for loading DNA into EVs. Under 2,000 V for 5 ms, the EVs showed positive fluorescent signals with Dil (Figure 4Biii) with a change of the structure in side scatter (Figure 4B) when compared with the control (Figure 4A). The positive signal of EVs with Picogreen suggested that these EVs were carrying DNA plasmid (Figure 4Cii). The double staining with both Picogreen and Dil showed that EVs could be loaded with DNA (DNA-EVs) and that electroporation was an effective loading procedure.

### Transfection of Extracellular Vesicles With THP-1 Cell Lines

**FIGURE 5:**
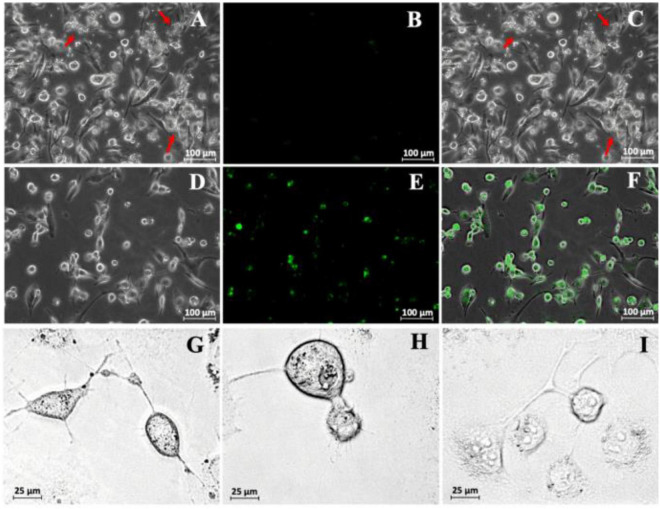
**(A)** Transfection of THP-1-derivated macrophage M2 with DNA-extracellular vesicles (Evs). A bright-field image was taken just after the infection of DNA-EVs. The red arrows indicate the DNA-EVs adhered together with macrophages and also on cell culture surfaces. **(B)** Fluorescence image with no green signal. **(C)** Overlay of bright-field and fluorescence images. Further images were taken after 36 h transfection: bright field **(D)**, fluorescence **(E)**, overlay **(F)**. Images to see the stretch of macrophage arms to capture EVs on the cell culture surfaces were taken after 6 h **(G)**, 12 h **(H)**, and 96 h **(I)** transfection.

To test the ability of the DNA-EVs to release their cargo at putative target cells, M2 macrophages derived from THP-1 cells (as macrophage M2) were transfected with DNA-EVs (Figure 5). There was no significant fluorescence signal of green fluorescent protein (GFP) observed except the autofluorescence of cells during the transfection time up to 96 h (Figure 5B). During the culture, macrophages attached to cell culture surfaces showed the amoeboid movement with stretching filopodia to engulf surrounding particles (Figure 5D). In addition, the ratio of dead cells stained with trypan blue was relatively low within 72 h under experimental conditions. However, after 96 h of transfection, some macrophages showed apoptotic signals, such as cytoplasmic vacuolization, swollen endoplasmic reticulum, and cell blebbing (Figure 5I). Although the expression of GFP was observed in a sub-population of cells, however, the expression was not stable. In many trials, the transfection efficiency was low and the expression was significantly reduced after 48 h (Figure 5E).

**FIGURE 6:**
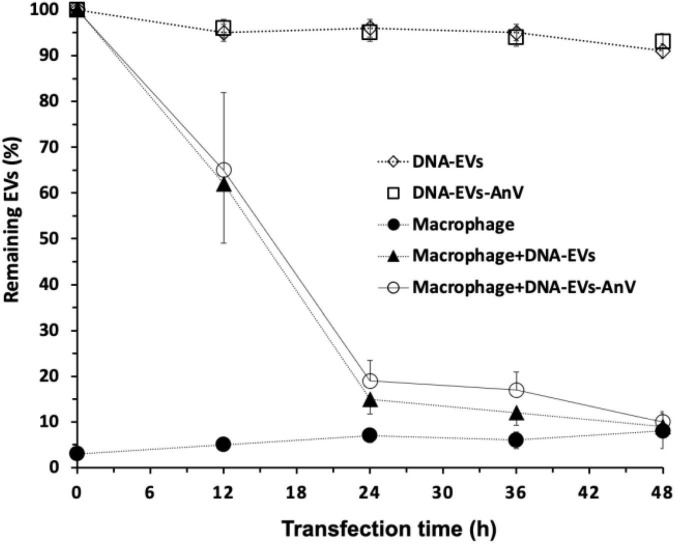
Remaining EVs after infection to THP-1 cells. **(↓)** DNA-extracellular vesicles (EVs; (EVs containing DNA plasmids) were stable within 48 h. (≤) DNA-EVs treated with Annexin V protein. (λ) THP-1-derivated macrophages M2. (π) DNA-EVs infected with macrophages M2. (Υ) DNA-EVs coated with Annexin V protein infected to THP-1 cells.

Although we could show that DNA-EVs can be taken up by the phagocytic active macrophages, it would be interesting to see if this uptake could be reduced by treating the EVs with annexin V protein. This should shield the “eat me signal” by the exposure of PS on the outer membrane leaflet of the EVs. As shown in Figure 6, the treatment with annexin V protein could not prevent the DNA-MV uptake by the macrophages, as the number of remaining EVs after different time intervals. The controls (DNA-EVs) were stable and slightly reduced within 48 h. However, after 12 h of transfection with macrophages M2, the number of EVs in the culture was reduced tremendously, the remaining DNA-EVs and DNA-EVs-AnV were about 60 and 62%, respectively. After 24 h, EVs were almost captured and engulfed by macrophages. There was no significant difference between the number of remaining DNA-EVs and DNA-EVs-AnV for all the time courses of transfection (*p* > 0.05; Figure 6).

## Discussion

During erythropoiesis, physiological cellular aging, disease conditions, and in response to environmental stressors, RBCs release EVs that include both endosome-derived exosomes and plasma-membrane-derived microvesicles (Thangaraju et al., 2020). Mature RBCs are anucleated cells without membrane-enclosed organelles. Therefore, RBC-EVs can be potentially used as a vehicle for DNA transport. It has been known that the formation of EVs is triggered by shedding of cell membrane caused by various factors, such as increased intracellular Ca^2+^ concentration, activation of PKC (Tissot et al., 2013; Nguyen et al., 2016; Thangaraju et al., 2020), disruption of erythrocyte skeleton-membrane attachment, aging-associated oxidative stress, ATP depletion (Asaro et al., 2018; Sudnitsyna et al., 2020), storage lesion (Leal et al., 2018; Prudent et al., 2018; Hashemi Tayer et al., 2019; Yoshida et al., 2019; Freitas Leal et al., 2020), and certain diseases (Regev-Rudzki et al., 2013; Jansen et al., 2017).

In the case of RBCs, the formation of EVs *in vivo* is believed as a homeostatic process to remove damaged cell constituents, such as oxidized hemoglobin and damaged membrane constituents, thereby prolonging their lifespan (Leal et al., 2018). However, it has been proved that RBC-derived EVs are not homogeneous in their size and content (Tissot et al., 2013; Marcoux et al., 2016; Freitas Leal et al., 2020). In this study, RBC-EVs were artificially produced by stimulating RBCs with PMA with a sufficient number within 2 h in a reproducible manner. Activation of PKC by PMA led to the formation and release of EVs from human RBCs and could be observed after 10 min treatment and the density of EVs significantly increased within 60 min (Figure 1). This process likely involved the activity of the PKCα and the TRPC6 channel (Wagner-Britz et al., 2013; Wang et al., 2021).

It has been known that mature RBCs do not have organelles, so RBC-EVs can be considered as the ideal vehicle for carrying and transporting macromolecules or therapeutic drugs to target cells. As RBCs are the most abundant cells in our body, representing above 80% of all the cells in an average adult human being (Kuo et al., 2017; Della Pelle and Kostevšek, 2021), therefore, it is relatively simple and rapid to prepare a large amount of EVs for research and treatment without requiring cell culture. In addition, this becomes more advantageous in the case of personalized medicine when the EVs can be isolated directly from individual patients. Currently, RBC-EVs are also provided by Cellarcus Biosciences Inc., as research materials with an average size of 140 nm (in the range from 80 to 210 nm). The Cellarcus’ human red blood cell-derived vesicles are prepared from RBCs induced by a Ca^2+^ selective ionophore. Vesicles are isolated from cells and debris *via* centrifugation and provided at a concentration of 10^7^ vesicles/μl.

At present, the classification of the subsets of EVs is still difficult due to the lack of EV-specific “markers” that enable to distinguish these subsets from each other (Lötvall et al., 2014; Witwer et al., 2017; Witwer and Théry, 2019). Because RBC-EVs are highly heterogeneous in size, morphology, and metabolomic components (Tissot et al., 2013; Lötvall et al., 2014; Murphy et al., 2019) and there is no currently “gold-standard” method to isolate or purify EVs. Therefore, in some cases, the isolated EVs may contain a mixture of different subsets of EVs (Cvjetkovic et al., 2014; Lötvall et al., 2014).

For a period of time, RBC-EVs have been thought to be cellular garbage sacs, however, various types of microRNAs were recently found in RBCs and their released EVs (Sun et al., 2020). Therefore, the roles of RBC-EVs could go far beyond the current understanding. However, in our study, the recognition and clearance of RBC-EVs by M2 macrophages (Figures 5, 6) suggested that the erythrophagocytosis by macrophages could be a barrier to the application of RBC-EVs in practice.

In the era of gene therapy, the delivery of nucleic acids, such as the small interfering RNA (siRNA), mRNA, or plasmid DNA in target cells, is a challenge with numerous implementations under development (Kaestner et al., 2015). Liposomes have been most widely applied as carriers because they are easy to synthesize and modify their characters to change their form, rigidity, permeability, retention, or conjugation with stealth polymers (Della Pelle and Kostevšek, 2021). Recently, the mRNA vaccines against severe acute respiratory syndrome coronavirus 2 (SARS-CoV-2) of Pfizer/BioNTech and Moderna were protected and delivered effectively to the target cells by 1,2-dimyristoyl-sn-glycero-3-phosphocholine liposomes. Doxorubicin (Doxil^®^ or Caelyx^®^) and amphotericin B (under commercial name AmBisome^®^, and MM-398^®^/Onivyde) were also capsulated by liposomes. However, artificial nanoparticles prepared by polymeric, dendrimeric, liposomal, or combinatory approaches are often recognized as non-self by the immune system (antibody or macrophages) (Della Pelle and Kostevšek, 2021). So far, it is difficult to synthesize inert nano-carriers, therefore, an effort to prepare nano-bio materials as vehicles for macromolecules or drug transport should be focused on. At present, most nucleic acid carriers have a cationic charge to maximize the stability of the formulation and to enhance cell delivery (Jiang and Thayumanavan, 2020), while there are some concerns about toxicity. Therefore, neutral or mildly anionic materials to entrap or carry nucleic acid have been started to be investigated, to ensure better biocompatibility, and broaden the application range (Della Pelle and Kostevšek, 2021).

Although liposome formulations remain the most used, cell-based drug-delivery systems, EVs could bring friendly vehicles or even be self-originated for personal medicine (Ryu et al., 2014). Using the vehicle structures from the body’s cells, as drug delivery systems is a promising strategy to overcomethe limitations of existing drug-delivery approaches. RBC ghosts have been studied for intracellular delivery of genetic materials, drug delivery as a vascular carrier (Muzykantov, 2010). As empty sacs, biotherapeutic agents can be encapsulated in and the cell membrane may serve as a surface to reduce immune reaction to these agents or protect from pathways of inactivation (Villa et al., 2016). The membrane of ghost cells can be considered, from the drug delivery system point of view, perfect stealth, biocompatible, highly elastic, and deformable made of phospholipids and cholesterol. As particles shed directly from the cell membrane and possess a small size at the nanoscale, spherical shape, surface potential, and membrane permeability, the vesicles can be exploited to deliver particular molecules by loading them *ex vivo* (Zhang et al., 2018; Zipkin, 2020). RBC-EVs draw the attention of the scientific community due to their ability to cargo nucleic acid, drugs, or biotherapeutic agents (Sun et al., 2020).

One of the focusing points for developing a reliable, stable drug delivery system is the loading of nucleic acid, drug, or bioactive compounds into the EVs. Recent studies showed that the exosome is an outstanding platform candidate for cargo delivery (Mandal, 2016; Maas et al., 2017; Usman et al., 2018; Gangadaran and Anh, 2020; Della Pelle and Kostevšek, 2021; Gutierrez-Millan et al., 2021). Various strategies have been developed for loading materials into exosomes, e.g., by incubation, the desired cargo can be diffused across membrane structures and packaged within exosomes (Fu et al., 2020; Han et al., 2021). Nucleic acids can be loaded into EVs by transfection-based strategy or physical treatments, such as electroporation, sonication, and surfactant treatment that facilitate cargo loading (Fu et al., 2020; Xu et al., 2020; Han et al., 2021). For the present study, electroporation at 2 kV for 5 s was suitable for loading of DNA plasmid to RBC-EVs (Figure 3). However, electroporation led to an increase of PdI that indicated the broad size distribution of DNA-EVs or contained large particles or aggregates (Figure 3C). Another study also proved that electroporation providing extra electrical fields produces micro-pores on the membrane of EVs to enhance permeability (Alvarez-Erviti et al., 2011). In our study, electroporation showed a simple and effective approach to load DNA plasmids into RBC-EVs that was proved by flow cytometry analysis with the fluorescent dyes Dil and Picogreen (Figure 4). Freeze-thaw treatment, extrusion, and dialysis may be applied to load cargo into EVs (Fu et al., 2020).

A general limitation of delivery systems is the recognition and elimination of foreign substances by the immune system. Biomimetic vehicles, such as cell membrane-derived carriers, may disguise cargo and escape from macrophage in the immune systems (Li et al., 2018; Sudnitsyna et al., 2020). CD47 was reported to involve the inhibition of phagocytosis of RBCs through the receptor signal regulatory protein alpha (SIRPα) (Hatherley et al., 2008; Kriebardis et al., 2008; D’Alessandro et al., 2010; Tissot et al., 2013; de Back et al., 2014). Another report also noted that CD47 did not only function as a “do not eat me” signal for uptake but can also act as an “eat me” signal (Burger et al., 2012). Therefore, it is questionable whether CD47 supports the escape of EVs from the clearance by macrophages. In the present study, the DNA-EVs were eliminated by macrophages M2 after 24–48 h transfection (Figure 5). In our study, although the signal of GFP was observed, the expression was observed in a subpopulation of cells. In addition, the expression was not constitutive and stable in many experiments, which might be due to the transient expression, low transfection efficiency, or the erythrophagocytosis. In addition, the mechanism of the DNA uptake by THP-1 cells is still the question. It could be either whole EV taken up in an endocytotic manner or/and the EVs fused with the membrane and showing a transient expression in a sub-population of M2 macrophages was still a question. In reports, the transfection to THP-1 cells was more effective *via* viral transduction, such as a process mediated by lentivirus or herpes simplex virus (Ferreira et al., 2019; Venuti et al., 2019), and in some cases, the electroporation (nucleofection) would be also preferred (Maeß et al., 2014). To date, the mechanism of DNA carried by EVs and gene expression in THP-1 cells after EVs interacted with these cells remains to be elucidated.

In our study, the engulfment and clearance of EVs by macrophages M2 required further investigation in future research. A study on the efferocytosis of exosomes derived from T-cells by monocytes explained the clearance of those exosomes by the PS-related pathway (Wei et al., 2016). Treatment with annexin V inhibited the uptake of apoptotic cells by macrophages due to masking the exposure of PS (Bennett et al., 1995). In a study in mice, the treatment of exosomes with annexin V protein significantly reduced the clearance of macrophages up to 66% (Matsumoto et al., 2017). However, in our study, there was only a slight reduction of the density of EVs with no significant difference between EVs treated and untreated with annexin V protein (*p* > 0.05) (Figure 6).

**FIGURE 7:**
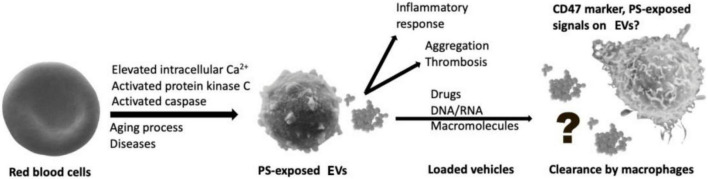
Macrophages act as a barrier to recognize and eliminate red blood cell-derived extracellular vesicles (RBC-EVs).

It is worthwhile to mention that in contrast to the aforementioned finding, our study was performed *in vitro*. *In vivo* and *in vitro* conditions represent completely different interaction scenarios (flow vs. stasis) and different surrounding media (blood plasma vs. cell culture medium). Therefore, we cannot present an uptake mechanism and its putative regulation, which will be the subject of further research. Although the recognition and uptake of exosomes by macrophages have been investigated, studies on the interaction between receptors of macrophages with membrane proteins, PS, and other lipids of EVs require a better understanding (Mulcahy et al., 2014; Birge et al., 2016; Naeini et al., 2020; Figure 7).

Therefore, further studies should be carried out to clarify the roles of EVs in the homeostasis of RBCs and cell communication. Although RBC-EVs are vehicle structures from the body’s cells, the use of these vehicles to cargo macromolecules for therapy or treatment is still a challenge. However, the GFP-coding plasmid resulted in a GFP expression in the macrophages. Although we have to admit that it was not the goal of our study, we could show that RBC-EVs in the way we designed them can be used as nucleic acid carriers if the immune system is addressed.

Further studies, e.g., using stable fluorescent dyes or radioactive isotopes to labeled DNA molecules, bioactive compounds, or drugs loaded into RBC-EVs are required to underline the fate of EVs and the delivery of cargos to the target cells. In addition, a study on the modification of RBC-EVs to hide from recognition and elimination of macrophages would contribute to developing cell-based systems for drug delivery or even self-originated for personalized medicine. In conclusion, the present study provides information for the physiological functions of RBC-EVs as well the potential for the application of these EVs as a cell-based delivery system.

## Data Availability Statement

The raw data supporting the conclusions of this article will be made available by the authors, without undue reservation.

## Ethics Statement

The studies involving human participants were reviewed and approved by the Research Ethics Committees of the Saarland University (approval 63/11). Written informed consent for participation was not required for this study in accordance with the national legislation and the institutional requirements. The animal study was reviewed and approved by the Research Ethics Committees of the Saarland University (approval 63/11).

## Author Contributions

DN and IB conceived the idea. DN and HT designed the study, wrote the first manuscript draft, and analyzed the data. LK and IB provided materials and support. All authors contributed to write the manuscript. All authors contributed to the article and approved the submitted version.

## Conflict of Interest

The authors declare that the research was conducted in the absence of any commercial or financial relationships that could be construed as a potential conflict of interest.

## Publisher’s Note

All claims expressed in this article are solely those of the authors and do not necessarily represent those of their affiliated organizations, or those of the publisher, the editors and the reviewers. Any product that may be evaluated in this article, or claim that may be made by its manufacturer, is not guaranteed or endorsed by the publisher.
